# Recent Advances in Materials, Synthesis, and Reaction Model of Particle Adsorbent for Flue Gas Desulfurization

**DOI:** 10.3390/molecules30081653

**Published:** 2025-04-08

**Authors:** Yanni Xuan, Kun Yu, Hong Tian, Zhangmao Hu, Wei Zhang, Yanshan Yin, Haitao Gao, Qingbo Yu

**Affiliations:** 1 School of Energy and Power Engineering, Changsha University of Science and Technology, Changsha 410114, China; xuanyanni@csust.edu.cn (Y.X.); a252003269@163.com (K.Y.); huzhangmao@163.com (Z.H.); weizhang@csust.edu.cn (W.Z.); yanshan.yin@csust.edu.cn (Y.Y.); 2 State Key Laboratory of Precision Manufacturing for Extreme Service Performance, Central South University, Changsha 410083, China; gaohaitao@csu.edu.cn; 3 School of Metallurgy, Northeastern University, Shenyang 110819, China

**Keywords:** particle adsorbents, active components, inert carriers, preparation techniques, gas–solid reaction models

## Abstract

Particle adsorbents have gained significant traction in flue gas desulfurization applications, primarily attributed to their high structural homogeneity and large specific surface area. To address the multifaceted requirements of industrial sectors regarding the structural configurations and physicochemical properties of particle adsorbents while promoting sustainable manufacturing practices, this study systematically evaluates and critically appraises contemporary advancements in particle desulfurizing agent technologies. The synthesis of these findings establishes a theoretical framework to facilitate technological innovation and industrial progress within the particle desulfurizer domain. The research systems of particle adsorbents, encompassing active components, inert carriers, preparation methodologies, and gas–solid reaction models, were comprehensively reviewed. The advantages and current limitations of these systems were then systematically summarized. Finally, the fundamental principles and research trajectories in the application fields of distinct particle adsorbent research systems were elucidated. An analysis of the developmental trends indicated that enhancing the utilization efficiency of active components and improving the cyclic stability of adsorbents remained critical engineering challenges. It is posited that the pursuit of high reaction activity, thermal stability, mechanical strength, and superior anti-aggregation/sintering performance constitutes key directions for the advancement of particle adsorbents in China’s flue gas desulfurization industry.

## 1. Introduction

With the escalating global environmental crisis, energy conservation and emission reduction have garnered significant scholarly interest. Low-concentration sulfur dioxide (SO_2_), as a hazardous air pollutant, poses substantial risks to ecosystems and human health [1], rendering it a critical research priority. Notwithstanding the substantial progress achieved through policy implementations such as the “Ten Measures for Atmospheric Pollution Control” and the “Three-Year Action Plan for Winning the Battle for a Blue Sky”, China’s total SO_2_ discharges remain among the highest globally due to ongoing industrialization and urbanization processes, exacerbating ecosystem degradation at critical levels [2]. Notably, the Ambient Air Quality Standard [3] establishes distinct regulatory frameworks for ambient air engineering zones. Class I areas, encompassing nature reserves and scenic regions, enforce the primary standard with a one-hour SO_2_ concentration limit of 150 μg/m^3^. Conversely, Class II areas—including residential, commercial–transportation–residential mixed, and rural zones—are subject to the secondary standard, which permits a one-hour concentration of 500 μg/m^3^.

To satisfy the increasingly stringent environmental protection emission standards, energy-efficient purification systems for low-concentration SO_2_ flue gas are pivotal for advancing green manufacturing and carbon neutrality. Conventional approaches such as the limestone–gypsum, magnesium oxide, and sodium alkali processes exhibit superior purification performance for low-concentration SO_2_ [4,5,6,7]. Nevertheless, these processes generate low-value byproducts including calcium sulfate, magnesium sulfite, and sodium sulfate. Upon improper disposal, these materials may induce secondary contamination and squander sulfur resources [8].

**Figure 1:**
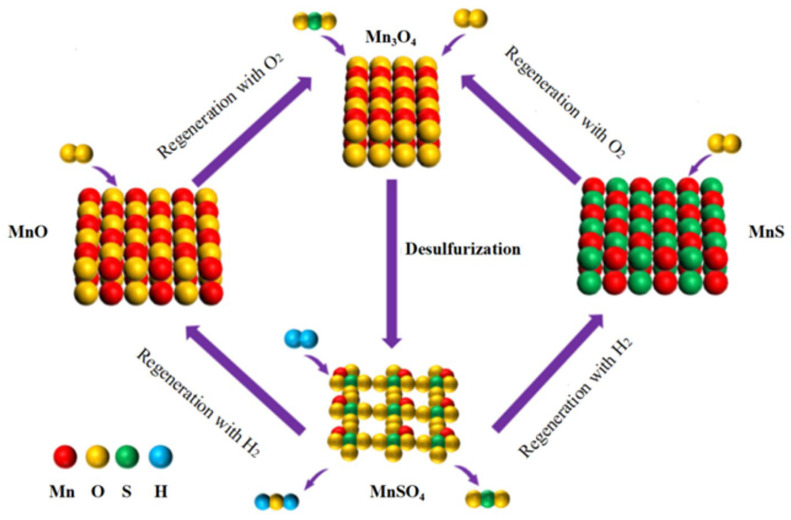
Chemical looping flue gas desulfurization process.

A novel flue gas desulfurization protocol, founded on chemical looping technology, was put forward by Sohn and Kim [9]. It was demonstrated that metal oxides serving as the desulfurizer could be recycled, and that sulfur resources could be efficiently collected during the regeneration process [10,11]. A flowchart of the chemical looping desulfurization procedure is presented in Figure 1. Therefore, to depict the chemical looping desulfurization process more precisely and quantitatively, the chemical looping flue gas desulfurization process of Mn_3_O_4_ [12], which comprises a desulfurization stage, a regeneration stage with H_2_, and a regeneration stage with O_2_, is elucidated as follows:

① Desulfurization Procedure: Mn_3_O_4_ serves as the active component of the adsorbent. Due to its exceptional sulfur-fixing capacity, Mn_3_O_4_ undergoes a reaction with SO_2_ in flue gas to form MnSO_4_, as shown in Equation (1) (E1).

(1) $${\mathrm{Mn}}_{3}O_{4}{+3\mathrm{SO}}_{2}{(g)+O}_{2}\left(g\right)\to {3\mathrm{MnSO}}_{4}$$

② Regeneration with H_2_ process: During this stage, MnSO_4_ is regenerated into MnS and MnO, with sulfur recovery occurring concurrently. The types of products are primarily influenced by the amount of H_2_ input. Initially (mH_2_:mMnSO_4_ < 1:1), MnSO_4_ is reduced to MnO (E2), and a significant amount of SO_2_ is released (Stage 1). Subsequently, as the H_2_ input increases (Stage 2, where 1:1 < mH_2_:mMnSO_4_ < 3:1), the residual SO_2_ in the reactor reacts with H_2_ to form elemental sulfur (E3). Finally, when the molar ratio of H_2_ to MnSO_4_ exceeds three (Stage 3), the residual elemental sulfur in the sorbent is reduced to H_2_S, which then reacts with MnO to form MnS (E4). Therefore, it is demonstrated that the optimal reaction pathway is to control the molar quantity of input H_2_ to be three-fold that of MnSO_4_ (E3). However, in practical experiments, the generation of a trace amount of MnS (E4) is unavoidable.

(2) $${\mathrm{MnSO}}_{4}{+H}_{2}\left(g\right)\to {\mathrm{MnO}+\mathrm{SO}}_{2}{(g)+H}_{2}O(g)$$

(3) $${\mathrm{MnSO}}_{4}{+3H}_{2}\left(g\right)\to {\mathrm{MnO}+S+2H}_{2}O(g)$$

(4) $${\mathrm{MnSO}}_{4}{+4H}_{2}\left(g\right)\to {\mathrm{MnS}+4H}_{2}O(g)$$

③ Regeneration with O_2_ process: During this stage, MnO is oxidized to Mn_3_O_4_ (E5). Concurrently, a trace amount of MnS is converted into Mn_3_O_4_ by reacting with O_2_ (E6). Thus, the aforementioned chemical looping flue gas desulfurization protocol completes one cycle.

(5) $${3\mathrm{MnS}+5O}_{2}\left(g\right)\to {\mathrm{Mn}}_{3}O_{4}{+3\mathrm{SO}}_{2}\left(g\right)$$

(6) $${6\mathrm{MnO}+O}_{2}\left(g\right)\to {2\mathrm{Mn}}_{3}O_{4}$$

However, increasingly stringent requirements have been imposed on the performance of particle adsorbents by this novel flue gas desulfurization method. Particle adsorbents are required not only to have high uniformity and high specific surface area but also to possess certain structural characteristics. Therefore, the detailed differences between particle adsorbents and ordinary powder adsorbents are as follows.

Firstly, in terms of physical properties, particle adsorbents are in a granular form with a larger particle size, typically on the millimeter scale [13]. Conversely, ordinary powder adsorbents are in a powdered form with smaller particle sizes, usually on the micrometer or even nanometer scale [14]. Particle adsorbents are characterized by a complex internal pore structure, including both coarse and fine pores. The coarse pores allow for the rapid entry of adsorbate molecules into the interior of the adsorbent, while the fine pores provide a larger adsorption surface area [15,16]. In contrast, traditional powder adsorbents, with their small particle size, have high adsorption activity, and their pore structure is relatively simpler [17]. Secondly, regarding mechanical properties, particle adsorbents are prepared through special processes such as compression molding and sintering, resulting in higher mechanical strength. As a result, they are less likely to break or wear during use. On the other hand, traditional powder adsorbents, being in a powdered form, have lower mechanical strength and are more prone to agglomerate or break during use [18,19]. Thirdly, in the aspect of applications, particle adsorbents are commonly utilized in fixed-bed or fluidized-bed reactors, which makes them convenient for loading and separation. Conversely, traditional powder adsorbents are often employed in packed-bed or suspension systems, yet they are more difficult to separate [20]. Particle adsorbents are suitable for scenarios where high adsorption capacity, reusability, and regeneration are required [21]. In contrast, traditional powder adsorbents are more appropriate for situations demanding rapid adsorption and high adsorption efficiency. Finally, in terms of regeneration and reuse, particle adsorbents, owing to their higher mechanical strength, are more easily regenerated and can be reused multiple times. In contrast, traditional powder adsorbents, due to their tendency to break and agglomerate during use, are more challenging to regenerate and have fewer opportunities for reuse [12].

Thus, particle adsorbents exhibit significant advantages over ordinary powder adsorbents, primarily manifested in the following aspects: (1) The active components of particle adsorbents are uniformly loaded onto the carrier surface, thereby enhancing the utilization efficiency of the adsorbent’s active components [22,23]. (2) Porous particle adsorbents possess a high specific surface area, augmenting both desulfurization performance and thermal stability [24,25]. (3) The controllable structure of particle adsorbents enables the precise regulation of the adsorbent’s morphological architecture, concurrently enhancing mechanical strength, mitigating active component agglomeration, and augmenting cyclic stability [26]. (4) The surface of particle adsorbents can be modified via diverse physical or chemical protocols to introduce specific functional groups or active sites. This modification strategy not only tailors the adsorbent’s surface properties according to distinct adsorption requirements but also improves its adsorption selectivity and affinity for target substances [10,27,28,29,30].

**Figure 2:**
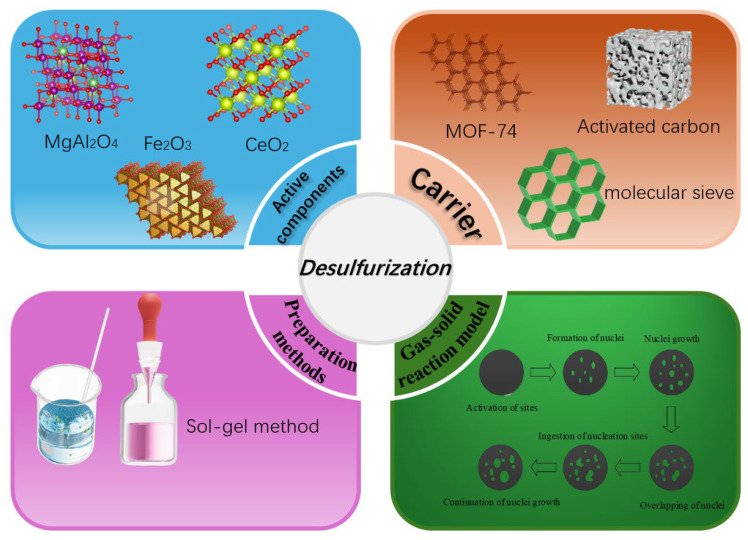
Active components, carrier, preparation methods and gas–solid reaction model of desulfurization.

For instance, diatomite and SBA-15 have been demonstrated to mitigate active component agglomeration and enhance utilization efficiency, which is attributed to their high surface roughness and porous architecture [27,31]. Substantial improvements in desulfurization efficiency were observed when incorporating the reactive components, surface functional groups, and catalytic/anti-agglomeration properties derived from porous Ca-based adsorbents [32]. Current investigations into particle adsorbents primarily center on the fabrication of composite materials, particularly metal oxide-inert carriers. However, given the pressing environmental protection requirements in high-tech contexts, there remains an imperative to advance the fundamental understanding of particle adsorbent systems [33]. Drawing on the development progress of common particle adsorbents, the research status of particle adsorbents for flue gas desulfurization is summarized in this article. Based on the construction process of active components and carriers with different compositions, as well as the preparation methods, an analysis is conducted. Subsequently, the reaction model of particle sorbents is further studied, as illustrated in Figure 2.

## 2. Active Components of Particle Adsorbents

### 2.1. Metal Oxides

During the preparation of particle adsorbents, the active components are identified as the key factors that restrict desulfurization performance. Notably, metal oxides are regarded as the most promising active components for particle adsorbents and are favored by numerous scholars [34,35,36,37]. This is because they exhibit high desulfurization efficiency, wide applicable temperature range, and high reaction activity. During the recycling of metal oxide adsorbents, several conditions must be satisfied. These include good adsorption potential, a minimal number of side reactions, weak reactivity with impurity gases, strong chemical stability, resistance to the loss of active components in a reducing atmosphere, high mechanical strength, excellent wear resistance, strong anti-pulverization ability, stable cycling performance, and being non-toxic, harmless, and environmentally friendly, etc. [38]. Currently, the mainstream particles predominantly focus on alkaline earth metal oxides (e.g., CaO, MgO), transition metal oxides (e.g., V_2_O_5_, CuO), rare earth metal oxides (e.g., CeO_2_, La_2_O_3_), and composite metal oxides [39,40,41,42,43,44,45,46]. Therefore, the sulfur -fixing potential and service conditions of various metal oxides are presented in Table 1.

**Figure 3:**
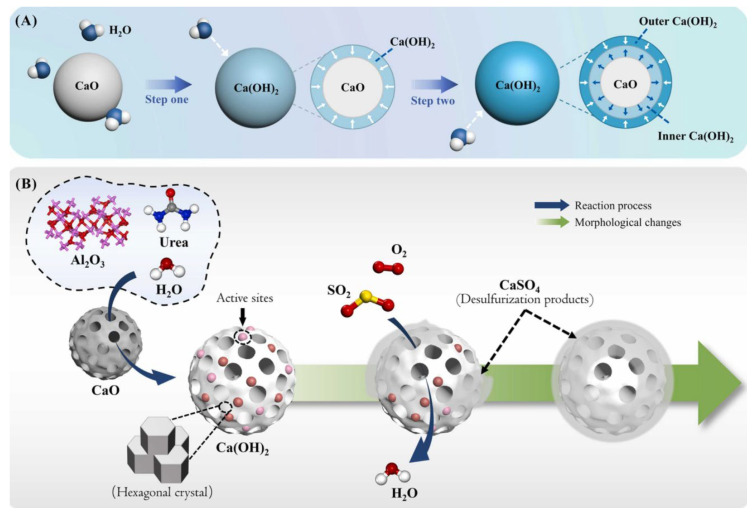
The illustration of (**A**) the lime digestion process and (**B**) the desulfurization process. Reprinted with permission form Ref. [54]. Copyright (2024) Elsevier.

Although various metal oxides exhibit promising adsorption capacities, substantial limitations have been documented. Calcium oxide (CaO), for instance, demonstrates superior adsorption efficiency in SO_2_ removal processes but suffers from irreversible sulfate formation during regeneration [54]. A calcium-based particle desulfurizer was synthesized by Xing et al. [54] via urea-assisted dissolution, yielding 92% sulfur capture efficiency under simulated flue gas conditions. The lime digestion process and the desulfurization process are shown in Figure 3. Copper and iron oxides, conversely, exhibit high initial desulfurization activities but undergo reductive deactivation in reducing atmospheres, as corroborated by thermodynamic modeling [49,50]. Nickel oxide sulfidation products further generate NiS during regeneration, leading to active site loss [52]. Rare earth metal oxides also demonstrate variable sulfur capture efficiencies depending on operating conditions [52]. Notably, manganese-based materials emerge as environmentally benign alternatives due to their radiation-free properties, sintering resistance, and cost-effectiveness. These oxides uniquely maintain stable sulfur fixation capacities without interference from CO_2_, H_2_O, or CO in flue gas. Mechanistic studies indicate that manganese oxides facilitate simultaneous denitrification reactions through redox interactions with NO. Furthermore, the hydrogenation of sulfided manganese oxides enables efficient SO_2_ recovery and elemental sulfur production under optimized conditions [47].

### 2.2. Doped Metal Oxides

To address the critical challenge of active component degradation during adsorbent preparation and application, contemporary research has focused on developing bimetallic composite materials. Previous investigations have demonstrated that rare earth metal oxides significantly enhance the structural stability and functional performance of adsorbents. Notably, Xia et al. [55] reported that La_2_O_3_ doping in Mn-based oxides reduces average crystallite size and improves manganese oxide dispersion on supports, thereby augmenting H_2_S removal efficiency. Furthermore, CeO_2_ has been shown to form strong synergistic interactions with metal oxides (e.g., CaO, MnOx), providing additional oxidation sites and enhancing surface desulfurization activity [56,57]. An investigation was conducted by Jae et al. [58] into dry MgO applications in petrochemical residue fluid catalytic cracking (RFCC) units. The desulfurizer was synthesized via coprecipitation, with Ce, Co, Fe, and Cu additives incorporated to enhance MgO properties. Sulfur removal reactions were performed at 700 °C, while regenerative processes occurred at 530 °C. Mathieu et al. [59] synthesized an MCM-41 adsorbent containing CuO, CeO_2_, and LiCl for SO_2_ capture from flue gas at 673 K. This composite exhibited superior adsorption performance, achieving a maximum capacity of 130 mg SO_2_/g adsorbent. Li et al. [60] employed impregnation methods to modify MnO_2_ with alkali metal ions, aiming to improve SO_2_ capture efficiency. Thermogravimetric analysis was used to evaluate composite performance, and the effects of alkali doping on MnO_2_ were systematically investigated. The results indicate that alkali metal doping enhances SO_2_ capture capacity, with the LiOH-doped (2.0 mol/L) composite demonstrating optimal performance (124 mg SO_2_/g material), representing an 18% improvement over pure MnO_2_.

### 2.3. Special Metal Oxide Configurations

Special metal oxide configurations such as spinel oxide and perovskite oxide can also be chosen as desulfurizers. As active components of particle adsorbents, spinel and perovskite offer remarkable advantages over traditional metal oxides. First, both possess rigid crystal structures that can resist high temperatures and prevent sintering, enabling them to keep a large specific surface area and numerous active sites [61]. Second, by selecting different metal ions or doping, the surface acidity–basicity, redox ability, and oxygen vacancy concentration can be precisely adjusted, which in turn enhances the adsorption and conversion efficiency of SO_2_ [62]. Third, their stable structures can notably suppress sulfation reactions. Moreover, their activity can be restored through regeneration, indicating excellent cycle performance [63]. In contrast to the limitations of metal oxides in medium-low temperature and low-sulfur situations, spinel and perovskite are more suitable for complex working conditions with high temperature and high sulfur content, showing great potential for efficient and long-lasting desulfurization. Zhao et al. [64] investigated the adsorption kinetics and regeneration kinetics of spinel-type MgB_2_O_4_ (B = Fe, Al) adsorbents. The SO_2_ adsorption kinetic equation indicates that in the adsorption process, gas hourly space velocity, temperature, adsorption ratio, and SO_2_ concentration are key factors affecting the adsorption reaction rate, while the effect of oxygen concentration is relatively minor. Song et al. [65] utilized converter sludge and desulfurization ash as raw materials to synthesize calcium ferrate while simultaneously removing harmful elements in a high-temperature environment. The research indicated that under a nitrogen atmosphere at 1100 °C, the main reaction products of desulfurization ash and converter slag were CaFe_3_O_5_ and Ca_2_Fe_2_O_5_. As the temperature increased from 800 °C to 1100 °C, the sulfur removal rate rose from 62.15% to 98.48%.

### 2.4. Non-Metallic Components

**Figure 4:**
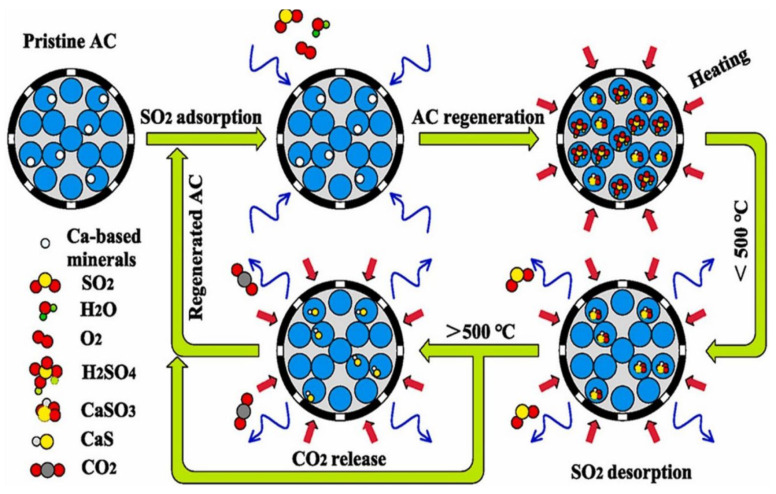
Thermal regeneration process and mechanism of activated carbon. Within the diagram, the green arrow denotes the reaction process, the blue arrow represents the gas adsorption/desorption process, and the red arrow signifies the heat in-put process. Reprinted with permission form Ref. [66]. Copyright (2022) Elsevier.

Non-metallic materials such as activated carbon (AC) are ubiquitously employed as support, concurrently facilitating sulfur dioxide (SO_2_) adsorption. Li et al. [66] systematically characterized the synergistic impacts of alkali/alkaline earth metal species in ash matrices on sulfur migration and carbon consumption via fixed-bed adsorption combined with thermal regeneration protocols. Significantly, their results demonstrated that the adsorption atmosphere exerted profound effects on gas desorption dynamics. Over the 150–850 °C temperature span, SO_2_ was identified as the primary contributor to excessive CO_2_ liberation during regeneration compared to non-adsorbed AC. Specifically, carbon loss observed in the lower temperature regime (150–500 °C) was primarily attributed to chemisorbed SO_2_ regeneration reactions. The thermal regeneration processes and mechanisms of AC are schematically illustrated in Figure 4. Within the diagram, the green arrow denotes the reaction process, the blue arrow represents the gas adsorption/desorption process, and the red arrow signifies the heat input process. Zhao et al. [67] conducted comprehensive investigations into the physical adsorption mechanisms of SO_2_ on edge-functionalized nanoporous carbons. Their findings demonstrated that the introduction of acidic oxygen-containing moieties or basic nitrogen-containing groups could significantly enhance SO_2_ physisorption capacities. Concurrently, Sheng et al. [68] reported the synthesis of nitrogen-doped activated carbon via the co-activation of KSCN and KOH using hydrothermal lignin as a precursor. The resulting adsorbent exhibited a record-high specific surface area of 3504 m^2^/g and contained 3.37% nitrogen derived from KSCN, which synergistically improved its desulfurization efficiency. At 273 K, the maximum adsorption capacities for SO_2_ and benzene were determined to be 2260.68 mg/g and 1668.90 mg/g, respectively.

In summary, metal oxides as active components of adsorbents have advantages such as high desulfurization potential, high reactivity, and high cycling stability. However, different metal oxides cannot simultaneously possess the various reaction properties that desulfurizers should have. Therefore, screening suitable metal oxides as active components of desulfurizers is a prerequisite for preparing efficient desulfurizers.

## 3. Carrier of Particle Adsorbents

Pure metal oxides exhibit inherently limited specific surface area and inadequate thermal stability. Despite the introduction of pore-forming agents, the augmentation of specific surface area remains marginal. The adsorbent surface constitutes the primary locus for active component desulfurization [41,69]. Enhanced active surface area facilitates the establishment of sufficient diffusion channels and reaction spaces for reactive gases, thereby improving desulfurization efficiency and regeneration performance. The active components are loaded onto inert carriers with a certain specific surface area and pore volume. Consequently, the specific surface area of the adsorbent is increased, and the diffusion of gases and the utilization of the active components are promoted [70,71]. Notably, the mechanical strength of the adsorbent is improved, its thermal stability is enhanced, and the abrasion during the multiple sulfidation and regeneration processes of the adsorbent is reduced [72,73].

Therefore, supported adsorbents have emerged as a prominent area of research in the contemporary field of adsorbent studies. These adsorbents are typically fabricated by loading metal oxides onto inert carriers via diverse preparation protocols. Notably, common varieties of inert carriers encompass activated carbon, activated alumina (γ-Al_2_O_3_), TiO_2_, spinel, molecular sieves, mesoporous silica, diatomite, and bentonite, etc. [41,69,70,71,72,73,74,75,76,77,78,79,80,81]. The physical characteristics of these carriers are presented in Table 2.

### 3.1. Silicon-Based Carrier

**Figure 5:**
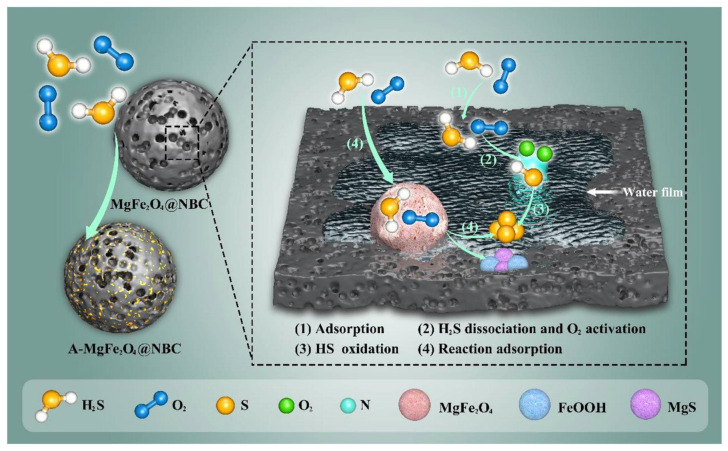
Proposed mechanism of H_2_S reactive adsorption and catalytic oxidation over the MgFe_2_O_4_@NBC. Reprinted with permission form Ref. [88]. Copyright (2023) Elsevier.

Despite the well-established industrial production processes of these carriers, significant variations in specific surface area, pore volume, and mechanical strength result in distinct carrier types for supported adsorbents, thereby exerting substantial effects on sulfur capacity and cycling performance. Silicon-based carriers such as diatomite, molecular sieves, mesoporous silica, and SBA-15 have been widely adopted in adsorption materials due to their advantageous physical properties, including high porosity, fine particle size, low thermal conductivity, lightweight characteristics, and large surface area [82,83,84,85]. Xuan [86] systematically evaluated the desulfurization performance of silicon carbide, diatomite, zeolite molecular sieve, and SBA-15. The SiC carrier exhibited a smooth surface with negligible internal porosity, leading to limited specific surface area for the desulfurizer; consequently, flue gas interactions were confined to active components on the carrier’s surface layer. In contrast, diatomite achieved 96% desulfurization efficiency, attributed to its favorable structural features and optimal active component dispersion. Zeolite molecular sieves, characterized by a unique three-dimensional pore architecture, enabled comprehensive flue gas penetration across internal and external surfaces, yielding a high sulfur capacity of 235 mg SO_2_/g adsorbent. However, SBA-15 demonstrated suboptimal desulfurization efficiency (88%), due to lower SO_2_ capture rates compared to the other tested materials. Zhang et al. [87] investigated xCuyMn/SBA-15 adsorbents for thermal coal gas desulfurization at 700–850 °C. The incorporation of thermally stable Mn_2_O_3_ active components onto SBA-15 significantly enhanced sulfur capacity at elevated sulfidation temperatures. Yuan et al. [88] developed a novel MgFe_2_O_4_-loaded N-doped biochar via a one-pot pyrolysis protocol using nitrogen-containing g-C_3_N_4_, waste rice, and metal salts, achieving efficient low-temperature H_2_S desulfurization. The adsorption and catalytic oxidation mechanisms are illustrated in Figure 5.

### 3.2. Metal Oxide Carrier

**Figure 6:**
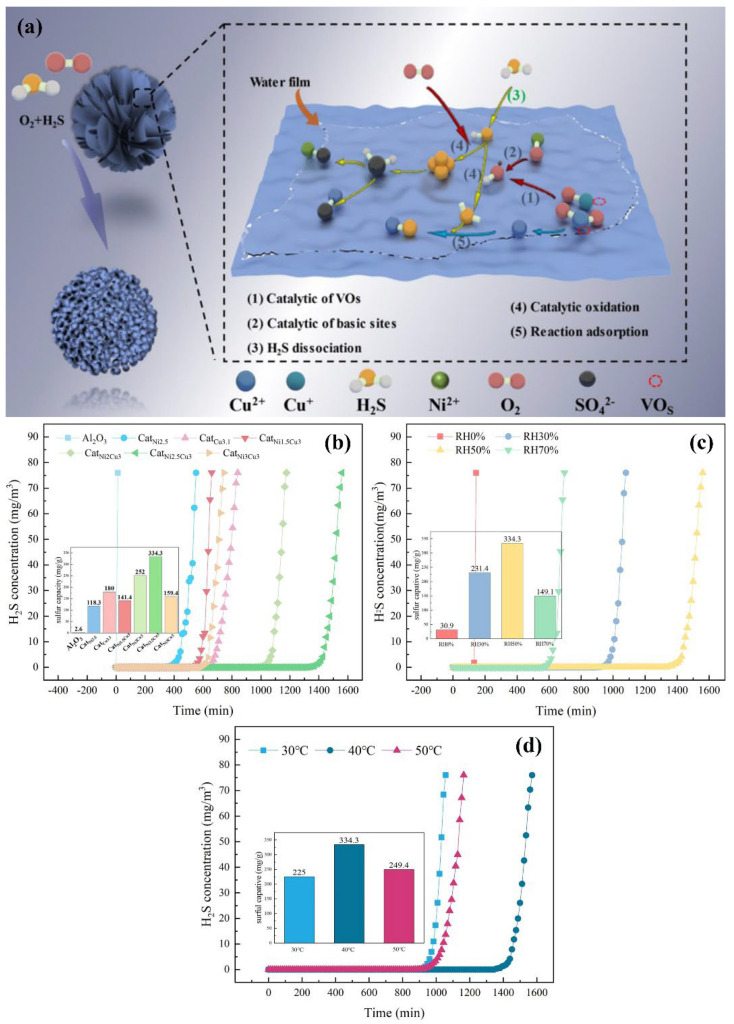
(**a**) Schematic diagram of desulfurization process of CatNixCuy catalyst; The breakthrough curves for catalytic oxidation of H_2_S: (**b**) at 40 ◦C and relative humidity 50%; (**c**) at different relative humidities; (**d**) at different temperatures. Reprinted with permission form Ref. [89]. Copyright (2023) Elsevier.

Yin et al. [89] synthesized a series of Ni-doped CuO_x_/Al_2_O_3_ catalysts using the surplus-volume impregnation method and studied their H_2_S removal performance at different temperatures and humidities. The doping of Ni can interact with Al_2_O_3_ and change the structure of the catalyst. At 2.5 wt% Ni content, 40 °C, and 50% relative humidity, the maximum H_2_S breakthrough sulfur capacity was achieved (334.3 mg/g). The H_2_S breakthrough curves of the prepared catalysts and desulfurization process are shown in Figure 6. Xu et al. [90] presented a novel Z-type heterostructure synthesized via a facile hydrothermal method, demonstrating exceptional oxidative desulfurization performance for model fuels. The optimized 7.5%Cu-ZnO/TiO_2_ catalyst achieved an 88.12% desulfurization efficiency within 240 min, attributed to its high specific surface area, abundant oxygen vacancies, and efficient charge separation enabled by the Z-scheme heterojunction architecture. The composite retained 80.69% efficiency after five cycles, implying its high stability. Song et al. [91] fabricated a low-cost activated carbon catalyst modified by Fe_2_O_3_ via the wet impregnation method. This catalyst was applied to the low-temperature co-catalytic reduction of CO, SO_2_, and NO. When Fe_2_O_3_ was 10% (by mass), the removal efficiencies of NO and SO_2_ reached 95% and 100%, respectively. Peng et al. [92] prepared a CaO/TiO_2_-Al_2_O_3_ adsorbent with a core–shell structure by a self-assembly template synthesis method. This core–shell structure effectively inhibited the reaction between the active component and the carrier, enabling it to exhibit excellent reaction activity, thermal stability, mechanical strength, anti-agglomeration and sintering performance during the cycling process.

### 3.3. Metal–Organic Framework Carriers

Metal–organic frameworks (MOFs) have emerged as a prominent area of investigation in carrier research over recent years. In general, the following three primary strategies enable MOFs to enhance toxic gas separation: (i) The generation of open metal sites can realize reversible coordinative binding processes and high gas sorption selectivities under ambient conditions. (ii) Ligand functionalization by functional groups such as highly electronegative fluorine atoms contributes to the strong charge-induced dipole interaction or hydrogen bonding between MOF and these toxic gases at low gas concentrations. (iii) Pore size adjustment is highly effective in enhancing the separation performance of toxic gases based on weak van der Waals interactions [93]. Ahmed and Jhung [94] introduced a novel tungsten oxide (WO_3_) catalyst supported on defective NU-1000 metal–organic framework (W@NU-1000) for efficient room-temperature oxidative desulfurization. The catalyst achieved near-complete conversion of dibenzothiophene (1000 ppm) within 120 min at 25 °C, with a low activation energy of 25.9 kJ/mol, attributed to well-dispersed WO_3_ species activating H_2_O_2_ and the MOF’s high porosity. The material demonstrates excellent recyclability, maintaining 99% activity over five cycles after simple acetone regeneration, highlighting its potential for practical applications in deep desulfurization under mild conditions. Zhang et al. [95] introduced a Zr-O cluster post-modification method in a Zr-MOF (MOF-808) to form EDTA-MOF-808 (EDTA, ethylene diaminetetraacetic acid) for selective and durable removal of SO_2_. The introduction of EDTA not only improved their SO_2_ adsorption capacity but also sharply increased their SO_2_/CO_2_ selectivity (57.2 vs. 8.9) and SO_2_/N_2_ selectivity (1915.8 vs. 292.7) at low SO_2_ partial pressure. Li et al. [96] integrated phosphotungstic acid (HPW) into Cu-based metal–organic frameworks (Cu-MOFs) using a hydrothermal synthesis method, aiming to address challenges in dry desulfurization such as slow reaction rates and low efficiency. HPW enhanced the catalytic oxidation of SO_2_ to SO_3_ through surface oxygen species, increasing SO_3_ content by 10.52% compared to pure Cu-MOFs. After four cycles, desulfurization efficiency remained at 76.4%, highlighting its recyclability. Xing et al. [97] encapsulated PMoV_2_ in four MOFs (MIL-101, HKUST-1, UiO-67, ZIF-8) with different cavity sizes and window sizes using a hydrothermal synthesis method. Then, a layer of mesoporous silica was coated on the surface of the MOFs to obtain four designed three-layer encapsulated catalysts 30%PMoV_2_@MOF@mSiO_2_.The results indicated that under simulated fuel of 2400 ppm, 30%PMoV_2_@UiO-67@mSiO_2_ had the highest desulfurization activity. After ten cycles, the efficiency could still reach 86.24%.

In conclusion, different carrier structures can inhibit the agglomeration of metal oxides and enhance the desulfurization and anti-sintering performance of adsorbents. Therefore, constructing composite metal oxide adsorbents with carrier structures can strengthen the structural stability of the adsorbents and intensify their cycling performance.

## 4. Preparation Methods of Particle Adsorbents

During the practical application of adsorbents, they are required to undergo repeated circulation between the desulfurization reactor and the regeneration reactor. Therefore, to ensure the excellent integrity and high reactivity of the adsorbents throughout the cycling process, the investigation of the components and structure of the adsorbents holds significant research value. Generally, the activity and structure of the adsorbents are closely correlated with the preparation methods. As presented in Table 3, a comparative analysis of different preparation methods and characteristics of the adsorbents was conducted [98,99]. The preparation of the adsorbents encompassed the processes of precursor preparation, drying, and calcination. Based on the distinct methods of precursor formation, they can be classified into the solid-phase method and the liquid-phase method.

The solid-phase methods predominantly encompass the mechanical mixing method and the freezing granulation method [100,101]. In the mechanical mixing method, the metal oxide powder and the inert carrier are first uniformly blended. Subsequently, deionized water is added for mechanical grinding to form a viscous substance, which is then pressed into a desired shape. Finally, the shaped sample undergoes drying and calcination processes [100]. Conversely, the freezing granulation method commences with the uniform mixing of the metal oxide powder, the inert carrier, a small quantity of dispersant, graphite or starch, and deionized water. Thereafter, the mixture is ground into a slurry in a ball-mill, sprayed into liquid nitrogen, and stirred to yield frozen granular products. Eventually, the adsorbent is prepared through freeze-drying and vacuuming procedures [101].

The liquid-phase methods primarily encompass the impregnation method, the sol–gel method, the dispersion method, and the coprecipitation method [102,103,104,105]. The impregnation method starts by impregnating the nitric acid solution of the active component onto the inert carrier and drying to prepare the precursor; then calcining the precursor nitrate to obtain the adsorbent [102]. The sol–gel method involves solidifying the nitric acid solution of the active component through sol–gel processes, followed by low-temperature drying and calcination to prepare adsorbents with molecular or nanostructures [103]. The dispersion method is relatively simple. It prepares the adsorbent by mixing, stirring, drying, and calcining the corresponding nitric acid solutions of the active component and the carrier [104]. The coprecipitation method involves adding an appropriate precipitant to multiple soluble solutions to prepare the precursor precipitate, then drying and calcining the precipitate to obtain the adsorbent [105].

**Figure 7:**
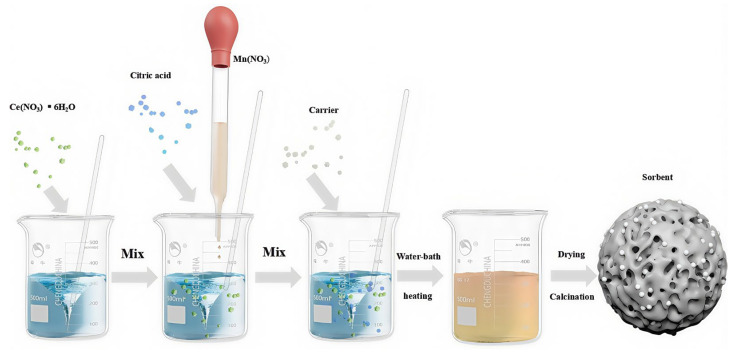
The preparation process of particle adsorbent by sol–gel method.

Among the traditional preparation methods of adsorbents, the mechanical mixing method and the impregnation method have simple operations and controllable components. However, the prepared adsorbent samples have poor uniformity and a limited loading amount of the active component, making it difficult to guarantee the activity of the adsorbent [100,102]. The dispersion method and the freezing granulation method improve the content of the active component of the adsorbent to a certain extent, but the microstructure of their adsorbents is difficult to control, the operation is complex, the preparation cost is high, and they are not suitable for large-scale applications [101,104]. The adsorbents prepared by the chemical coprecipitation and sol–gel methods have adjustable structures and can prepare high-performance nanostructured particles in Figure 7, but the preparation period of the adsorbents is long [103,105].

In conclusion, different preparation methods affect the reactive activity and cycling stability of the adsorbent. Choosing an appropriate preparation method to prepare an effectively controllable adsorbent structure and reaction efficiency is a necessary condition. Therefore, the preparation methods of adsorbents should develop towards high efficiency and fine control.

## 5. Gas–Solid Reaction Model

The dry flue gas desulfurization process is a typical gas–solid two-phase reaction. Therefore, it is of great significance to carry out research on the gas–solid reaction kinetics of adsorbents. Gas–solid reactions can be divided into gas–solid phase non-catalytic reaction processes and gas–solid phase catalytic reaction processes, and the dry flue gas desulfurization process belongs to gas–solid non-catalytic reactions. The gas–solid non-catalytic reaction process can usually be divided into the following steps [42]: (1) First is the diffusion of gaseous reactants from the gas phase to the surface of solid particles, that is, the external mass transfer step. (2) Second is the diffusion of gaseous reactants through the pores within the particles to the interior of the particles, that is, the internal diffusion step. (3) Third is the adsorption of gaseous reactants into active intermediate products or products, that is, the interface reaction step. The above steps occur simultaneously, and the link with greater resistance becomes the limiting step of the gas–solid reaction. In order to better understand the kinetic characteristics of gas–solid reactions, researchers have established corresponding gas–solid reaction models based on the characteristics of different types of redox reactions to describe the dependence of the conversion rate of adsorbents on time and the influence of different reaction conditions on the conversion rate of adsorbents. Currently, the mathematical models used to describe gas–solid reactions based on mechanism assumptions are mainly divided into shrinking core models, chemical reaction models, nucleation and nucleus growth models, and diffusion models. All gas–solid reaction models can be uniformly expressed as follows [106]:

(7) $$g(\alpha )=\int_{0}^{\alpha }\frac{d\alpha }{f(\alpha )}=\alpha^{m}{\left(1-\alpha \right)}^{y}{\left(-\ln(1-\alpha )\right)}^{p}$$

where g(α) is the integral form of the mechanism model function; f(α) depends on the reaction type or reaction mechanism and is the differential form of the mechanism model function; α is the conversion rate at a certain moment, %; m, y and p are constants, and any model can be obtained by assigning values to these three parameters.

For gas–solid reactions comprising multiple sequential steps, the controlling step dictates the overall reaction progression. Diverse geometric configurations of solid particles and distinct rate-controlling steps lead to varied kinetic model expressions. The common kinetic expressions of gas–solid reaction models [42] are summarized in Table 4.

### 5.1. Shrinking Core Model

**Figure 8:**
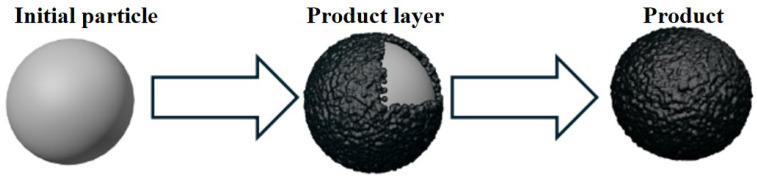
Reaction process for the shrinking core model.

The shrinking core model is primarily applicable to scenarios where the diffusion resistance of gas within unreacted particles is substantial. Initially, the reaction initiates at the outer surface of solid particles. As the reaction progresses, a product layer develops around the unreacted core, consequently causing the radius *r*_0_ of the unreacted core to gradually shrink (Figure 8). Cylindrical particle adsorbents are applicable to the cylindrical symmetry model (A_2_), while solid particles in spherical or cubic shapes correspond to the spherical symmetry model (A_3_). The active component in flue gas desulfurization adsorbents typically assumes spherical or cubic configurations [107], which can be mathematically described as follows:

(8) $$1-{\left(1-\alpha \right)}^{1/3}=kt/r_{0}^{\prime }$$

where k is the reaction rate constant; and $r_{0}^{\prime }$ is the radius of the solid particle at time $t_{0}$; *t* is the reaction time. Ishida et al. [108] employed the shrinking core model in the early stage to investigate the chemical looping combustion process of nickel-based oxygen carriers using hydrogen as fuel. Similarly, Lu et al. [109] demonstrated that the core-shrinking cylindrical boundary control model effectively captured the calcination dynamics of magnetite during chemical looping methane reforming.

### 5.2. Nucleation and Nucleus Growth Model

**Figure 9:**
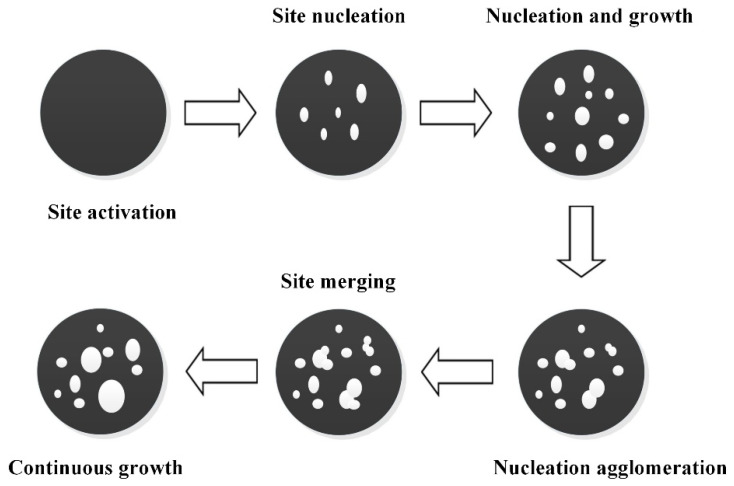
Reaction process for the nucleation and growth model.

The nucleation and growth model is applicable to redox reactions producing solid products [110]. In this process, nucleation sites are initially formed within particles, followed by progressive growth and overlap of these nuclei. During the reaction’s early stage, the reaction rate accelerates as the number of nucleation sites increases, a phase referred to as the induction period. Subsequently, the reaction proceeds uniformly across the solid surface (Figure 9). The nucleation and growth model is mathematically described by Equation (9), where the parameter h quantifies the relationship between nucleus growth and dimensionality. R_1_ denotes that nucleation sites are instantaneously formed and undergo one-dimensional linear growth; R_2_ indicates that nucleation sites are instantaneously formed followed by planar expansion; R_3_ represents that nucleation sites are instantaneously formed and then experience three-dimensional isotropic growth in three-dimensional space. Zhang et al. [111] demonstrated that the process of the Fe_2_O_3_/Al_2_O_3_ oxygen carrier in the chemical looping combustion of ultra-low concentration methane adheres to the nucleation and growth model. Moreover, the reduction of CoO to Co also follows the nucleation and growth mechanism [112].

(9) $${\left[-\ln(1-\alpha )\right]}^{1/h}=kt$$

### 5.3. Diffusion Model

**Figure 10:**
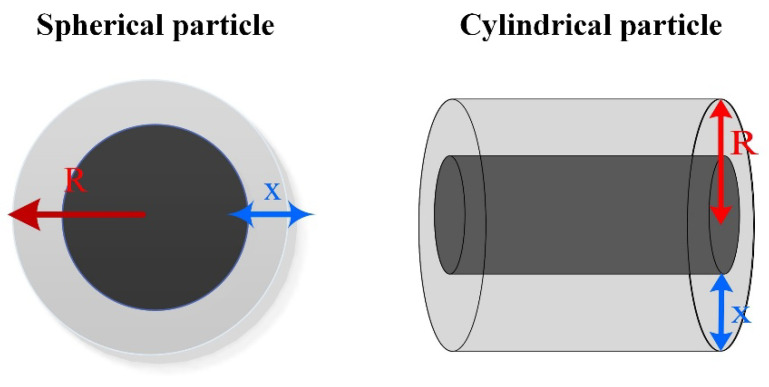
Reaction process for the diffusion models.

In diffusion-controlled reactions, the generation rate of solid products will decrease proportionally with the thickness of the product layer. According to the differences in diffusion dimensions, it can be classified into one-dimensional diffusion, two-dimensional diffusion, and three-dimensional diffusion. Based on the shape of particles, it can be further categorized into three-dimensional cylindrical diffusion and three-dimensional spherical diffusion. Under normal conditions, the solid particles used in gas–solid reactions are mainly spherical or cylindrical in shape. Therefore, the three-dimensional diffusion spherical symmetry and cylindrical symmetry models are the most widely used (Figure 10), shown as Equations (10) and (11), where *R* is the initial radius of the solid particle. The reaction process of trimanganese tetroxide and sodium carbonate is in line with the Ginstling–Brounshtein model in the diffusion model [113].

(10) $${\left(1-{\left(1-\alpha \right)}^{1/3}\right)}^{2}=\frac{kt}{R^{2}}$$

(11) $$(1-\frac{2}{3}\alpha -{\left(1-\alpha \right)}^{\frac{2}{3}})=\frac{kt}{R^{2}}$$

### 5.4. Chemical Reaction Model

The chemical reaction model is relatively simple and is similar to homogeneous kinetics. The concentration and quantity of reactants, as well as the amount of remaining reactants, will have a specific impact on the reaction rate, that is, the reaction order. For a 1.5-order reaction, the reaction occurs after the dissociative adsorption of gas molecules on the solid surface, where the adsorption process may act as the rate-controlling step, leading to a fractional order (1.5). A second-order reaction indicates that the reaction rate is proportional to the product of the concentrations of two substances or the square of the concentration of a single substance. Third-order reactions are relatively rare and typically involve either the simultaneous involvement of three molecules in the reaction or complex intermediate steps. The chemical reaction model can be expressed as Equation (12).

(12) $$\frac{d\alpha }{dt}=k{\left(1-\alpha \right)}^{n}$$

where *k* is the reaction rate; and *n* is the reaction order. The decomposition process of gadolinium complexes belongs to a zero-order chemical reaction model [114]. The high-temperature oxidation of porous silicon and the desorption process of 2-phenylethylamine belong to a first-order chemical reaction model [115].

Yang [116] fitted the flue gas desulfurization process data of particle adsorbents with the above gas–solid reaction model and determined that the flue gas desulfurization process using granular adsorbents follows a first-order reaction, with the gas–solid reaction kinetics described by a three-dimensional spherical diffusion model. The primary challenges in dry flue gas desulfurization with particle adsorbents are low desulfurization efficiency and slow reaction rates [117]. Kinetics focuses on exploring reaction rates, clarifying reaction mechanisms, and identifying rate-limiting steps in the process, where the controlling step directly impacts the reaction rate [118]. The gas–solid reaction process for flue gas desulfurization with particle adsorbents consists of the following three stages: external mass transfer, internal diffusion, and interfacial chemical reaction [119,120].

The gas–solid reaction in dry flue gas desulfurization with particle adsorbents is independent of interfacial chemical reactions, with the diffusion stage serving as the rate-controlling step [121]. During this process, the diffusion rate dictates the overall reaction rate. Therefore, improving desulfurization efficiency and reaction rates can be achieved by reducing diffusion resistance. The diffusion of gases in porous solids depends on the gas properties, the diffusion conditions, and the structure of the porous medium. In dry flue gas desulfurization with granular adsorbents, the prolonged residence time of SO_2_ gas in the pores of the adsorbent increases the likelihood of secondary reactions, degrading reaction selectivity. Reducing particle size to a certain extent can mitigate internal diffusion effects on the reaction, but excessively small particles may lead to entrainment and increased costs [122].

For reactions controlled by external diffusion, enhancing the overall process rate requires measures to accelerate external mass transfer, such as increasing the external surface area, improving gas flow properties, or elevating gas velocity. When SO_2_ molecules diffuse from the particle exterior into the internal pores of the adsorbent and the diffusion rate is slower than the interfacial chemical reaction rate, the reaction occurs in the diffusion-controlled region. Increasing the space velocity beyond a critical threshold can enhance diffusion rates, shifting the reaction to the kinetic-controlled region and eliminating external diffusion limitations. For flue gas desulfurization with particle adsorbents, optimizing space velocity, reducing particle size, and increasing pore diameters can minimize diffusion effects, thereby enhancing reaction rates and desulfurization efficiency [27].

## 6. Summary and Outlook

Particle adsorbents are an indispensable part of flue gas desulfurization systems. Improving the surface characteristics and physical properties of particle adsorbents, enhancing the utilization rate and stability of active components, and optimizing the adsorbent structure to meet multifunctional requirements and improve performance are important technical foundations for achieving ultra-low emissions. Currently, the research on particle adsorbents mainly focuses on optimizing the carrier structure and constructing bimetallic oxide composite adsorbents. However, large-scale application and theoretical research still face many challenges.

1. There are two core problems in the preparation and service of particle adsorbents: the active components are easy to agglomerate in the synthesis stage, and the crystallization phase growth and performance decline during recycling. Optimizing the carrier structure can improve the dispersion of active components, increase active sites and inhibit agglomeration. Although the core–shell structure can inhibit the reaction between the active component and the carrier, it is necessary to control the structural uniformity to maintain cycle stability. In the future, it is necessary to break through the synergistic reinforcement of rare earth doping on structure and performance, analyze the dispersion mechanism of active components, and solve the bottleneck of preparation and application.
2. Although the current preparation technology for particle adsorbents has achieved significant progress, there still exist a series of technical challenges such as poor uniformity, limited loading capacity of active components, difficult control of microstructure, complex operation, high preparation cost, and long preparation cycle. Particularly, under the premise of the national ultra-low emission standard, various industries have put forward even higher requirements for the microstructure and properties of particle adsorbents. In general, the preparation methods of adsorbents should develop in the direction of high efficiency and refined control.
3. The research system of particle adsorbents encompasses multiple disciplinary domains, including professional knowledge in fields like chemistry, materials science, and engineering. Developing a gas–solid reaction model that integrates multiple disciplines is highly conducive to promoting the optimization and upgrading of particle adsorbent preparation technology.

## Figures and Tables

**Table 1:** Desulfurization conditions of common metal oxides.

Metal Oxides	Desulfurization Efficiency/%	Experimental Temperature/°C	SO_2_ Concentration/vol.%	Flue Gas Flow/m^3^·h^−1^	Ref.
MnO_2_	95	200–250	0.07–0.17	0.12–0.15	[47]
CaO	83	600–800	0.2	0.06	[48]
CuO	96	350–450	0.12–0.5	0.05	[49]
Fe_2_O_3_	90	420–460	0.5–1	0.06	[50]
MgO	92	30–150	0.05–0.1	0.065	[51]
NiO	94	500–600	0.25	0.06	[52]
ZrO_2_	99	325	0.025	0.494	[53]
CeO_2_	82	500–600	0.25	0.06	[52]
Ni-Ce oxides	85	500–600	0.25	0.06	[52]

**Table 2:** Physical characteristics of inert carrier.

Carrier Name	Molecular Formula	The Characteristics of the Carrier	Ref.
Activated alumina	γ-Al_2_O_3_	The specific surface area is larger than 100 m^2^/g, the average pore diameter is 4–10 nm, the hydrothermal stability is relatively good, the applicable temperature range is wide, and it is prone to forming a spinel structure with metal components, which is not conducive to the regeneration of the adsorbent.	[41,69]
Titanium oxide	TiO_2_	The specific surface area is within 10 m^2^/g, the mechanical strength is high, and TiO_2_ has good resistance to SO_2_.	[70,71]
Spinel	MgAl_2_O_4_	The specific surface area ranges from 70 to 180 m^2^/g. The mechanical strength is higher than that of γ-Al_2_O_3_. It has stable properties, anti-sintering, wear-resistant, corrosion-resistant, impact-resistant, and has high strength and high hardness.	[72,73]
Zeolite molecular sieve	Na_12_Al_12_Si_12_O_48_	It has a three-dimensional framework structure. The specific surface area is larger than that of γ-Al_2_O_3_. The thermal stability and hydrothermal stability are high. However, it is not conducive to the preparation of adsorbents with high loading capacity, and the efficiency of the adsorbent is relatively low.	[74,75]
Mesoporous Silicon	SBA-15	It has hexagonal through-holes, and the pore size is adjustable. The relatively good thermal stability and large specific surface area increase the loading amount of metal oxides and result in a high utilization rate of the adsorbent.	[76,77]
Diatomite	SiO_2_	It has a macroporous porous structure (50–800 nm), a stable framework structure, high thermal stability, and the material is inexpensive. As an adsorbent carrier, it has great economic advantages.	[78,79]
Bentonite	Al_2_O_3_.4(SiO_2_)	It has a 2:1 type crystal structure composed of two silicon–oxygen tetrahedrons sandwiching one layer of aluminum–oxygen octahedron. It has strong hygroscopicity and expansibility and has an adsorption capacity for gases up to five times its own weight. It is a good adsorbent carrier.	[80,81]

**Table 3:** Preparation methods and characteristics of sorbents.

Method	Preparation Methods	Advantage	Disadvantage
Solid-phase method	Mechanical Mixing Method	The operation is simple, the conditions are easily controllable, and it is suitable for large-scale production.	The mixture is not uniform and the active components are prone to agglomeration.
	Freezing Granulation Method	The adsorbent has high activity and uniform particle size.	The preparation process is cumbersome with high cost and large energy consumption.
Liquid-phase method	Impregnation Method	The components are controllable. The content of the active components can be controlled by regulating the amount of the precursor solution.	The loading amount of the active components is controlled by the pore volume of the carrier.
	Sol–Gel Method	The dispersion of the active components is good; the microstructure is controllable; the specific surface area is high.	The raw materials are expensive and the preparation period is long.
	Dispersion Method	The dosage of the active components is precise; the mixture is uniform.	The demand for preparation raw materials is large and the process is complex.
	Coprecipitation Method	The particles are uniform and highly active; it is easy to obtain nano-scale adsorbents.	High local concentration causes the agglomeration of active components.

**Table 4:** Common functions of gas–solid reaction model.

Number	Gas–Solid Reaction Model	Differential Function	Integral Function
A_2_	Nucleus contraction (*h* = 2)	2 (1 − *α*)^1/2^	1 − (1 − *α*)^1/2^
A_3_	Nucleus contraction (*h* = 3)	3 (1 − *α*)^2/3^	1 − (1 − *α*)^1/3^
D_1_	One-dimensional diffusion	(1/2) *α*^−1^	*α* ^2^
D_2_	Two-dimensional diffusion	[−ln(1 − *α*)]^−1^	*α* + (1 − *α*) ln(1 − *α*)
D_3_	Three-dimensional cylindrical diffusion	(3/2) [(1 − *α*)^−1/3^ − 1]^−1^	1 − (2/3) *α* − (1 − *α*)^2/3^
D_4_	Three-dimensional spherical diffusion	(3/2) (1 − *α*)^2/3^[1 − (1 − *α*)^1/3^]^−1^	[1 − (1 − *α*)^1/3^]^2^
R_1_	Nucleation and growth (*h* = 1)	1 − *α*	−1*n*(1 − *α*)
R_2_	Nucleation and growth (*h* = 2)	2 (1 − *α*)[−ln(1 − *α*)]^1/2^	[−1*n*(1 − *α*)]^1/2^
R_3_	Nucleation and growth (*h* = 3)	3 (1 − *α*)[−ln(1 − *α*)]^2/3^	[−1*n*(1 − *α*)]^1/3^
C_3/2_	Chemical reaction (*h* = 1.5)	2 (1 − *α*)^3/2^	(1 − *α*)^−1/2^
C_2_	Chemical reaction (*h* = 2)	(1 − *α*)^2^	(1 − *α*)^−1^−1
C_3_	Chemical reaction (*h* = 3)	(1 − *α*)^3^	(1/2) [(1 − *α*)^−2^ − 1]

## Data Availability

No new data were created or analyzed in this study. Data sharing is not applicable to this article.
